# Increase in Cell Wall Thickening and Biomass Production by Overexpression of *PmCesA2* in Poplar

**DOI:** 10.3389/fpls.2020.00110

**Published:** 2020-02-20

**Authors:** Samaneh Sadat Maleki, Kourosh Mohammadi, Ali Movahedi, Fan Wu, Kong Shu Ji

**Affiliations:** Co-innovation Center for Sustainable Forestry in Southern China, The Key Forest Genetics and Biotechnology, Ministry of Education, Nanjing Forestry University, Nanjing, China

**Keywords:** *CesA*, cell wall, cellulose synthesis, plant growth, biomass production, poplar

## Abstract

Cellulose, the most abundant constituent material of the plant cell walls, is a major structural component of plant biomass. Manipulating cellulose synthesis (*CesA*) genes by genetic engineering technology, to increase cellulose production may thus offer novel opportunities for plant growth and development. To investigate this, here we produced transgenic “*Populus* 895 plants” overexpressing the cellulose synthase (*CesA2*) gene derived from *Pinus massoniana* under the control of constitutive 35S promoter, *via Agrobacterium*-mediated transformation. Relative expression levels of *PmCesA2* were functionally characterized in poplar hybrid clone “Nanlin895” (*Populus deltoides* × *Populus euramericana*). The results demonstrated the transgenic lines showed enhanced growth performance with increased biomass production than did the untransformed controls. It is noteworthy that the overexpression of *PmCesA2* in poplar led to an altered cell wall polysaccharide composition, which resulted in the thickening of the secondary cell wall and xylem width under scanning electron microscopy. Consequently, the cellulose and lignin content were increased. Hence, this study suggests that overexpression of *PmCesA2* could be used as a potential candidate gene to enhance cellulose synthesis and biomass accumulation in genetically engineered trees.

## Introduction

Plant cell walls are a terrifically important source of raw material for food, fuel, and industrially chemicals (Carroll and Somerville, 2009). In addition, they are responsible for not only conferring a definitive shape to the cell and enabling overall growth, but it also provides material transportation and protection of the cells' inner contents. Plant cell walls are rich in complex biopolymers carbohydrates that comprise cellulose, hemicellulose, and pectin (Daher and Braybrook, 2015) as well as lignin (Somerville et al., 2004). Cellulose, the linear (1→4)-β-D-glucan, is an important structural and functional component of both primary and secondary cell walls (Doblin et al., 2002). Plant primary wall surrounds growing and dividing plant cells while the secondary wall provides structural support to the xylem and the plant body (Scheller and Ulvskov, 2010; Zhong and Ye, 2015).

In most terrestrial plants, cellulose is thought to be synthesized at the plasma membrane by rosette cellulose synthase complexes which consist of cellulose synthase proteins (CESA) (Maleki et al., 2016). The *CesA* genes belong to membrane-bound glycosyltransferase family II (GT-2) enzymes (Sethaphong et al., 2013). These enzymes are characterized by two domains (A and B domains) which possess the D, D, and D, QxxRW motif, respectively (Saxena and Brown, 1997). The first plant *CesA* gene was successfully identified from cotton, by screening its expressed sequence tags (Pear et al., 1996). In *Arabidopsis*, at least three CesA subunits encoded by the *AtCesA1*, *AtCesA3*, and one of the *AtCesA6*-related genes (*AtCesA2*, *AtCesA5*, *AtCesA6*, or *AtCesA9*) are required for cellulose biosynthesis in primary cell walls, while *AtCesA4*, *AtCesA7*, and *AtCesA8* are required for secondary cell walls formation (Taylor et al., 2003; McFarlane et al., 2014). In aspen (*Populus tremuloides*), the three *PtrCesA1*, *PtrCesA2*, and *PtrCesA3* are highly expressed during secondary cell wall enriched xylem tissues (Joshi et al., 2004). *GhCesA* gene identification revealed that *CesA2* was a predominant gene for secondary cell wall formation (Li et al., 2013). Nairn and Haselkorn (2005) have shown that the phylogenetic and expression analysis of three loblolly pine *CesA* genes representing they are orthologous to the *CesA* genes in angiosperms which is responsible for cellulose synthesis in the secondary cell walls. These data suggested that these three genes *PtCesA1*, *PtCesA2*, and *PtCesA3* have been linked to secondary xylem development in gymnosperms *Pinus taeda*. Chu et al. (2007) observed that the knockout of *AtCesA2* caused severe defects in cell wall formation and microtubule orientation that led to abnormal plant growth and development. Furthermore, Chu et al. (2007) concluded that cellulose biosynthesis was needed for the microtubule orientation. It is noteworthy that microtubule orientation plays a critical role in controlling cell expansion and elongation. Correspondingly, mutations in *CesA* genes are known to loss of impair cellulose synthesis. For example, *cesa5/cesa6* double mutants were seedling lethal (Desprez et al., 2007), a mutation in *CesA6* -related genes (*CesA2*, *CesA5*, and *CesA9)* revealed only a mild phenotype (Scheible et al., 2001; Cano-Delgado et al., 2003) and *cesa2/cesa6/cesa9* triple mutants showed pollen lethality (Persson et al., 2007).

*Populus* plants are versatile and semi-evergreen forest trees with a wide distribution in northern China (Li et al., 2018). Their ease and simplicity of clonal propagation, rapid growth, and small genome size have made *Populus* tree species a well-established model organism for woody plant research (Bradshaw et al., 2000; Cai et al., 2017; Wang et al., 2018). Although the functions of CesA proteins are well studied in plants (Arioli et al., 1998), their genetic manipulation to enhance cellulose production, especially in timber trees, has remained demanding. For example, earlier overexpression of *CesA* genes has not resulted in improved plant growth in *Arabidopsis*, barley, and poplar (Zhong et al., 2003; Joshi et al., 2011; Tan et al., 2015). In this work, the *pBI121:CesA2* binary vector was constructed and introduced to the poplar hybrid clone “Nanlin895” (*Populus deltoides* × *Populus euramericana*) *via* the *Agrobacterium-*mediated transformation system. We found that *PmCesA2* overexpression in *Populus* influenced its secondary cell wall thickening as well as morphological and physiological traits. Analysis of molecular and morphological data and chemical composition of cell walls in transgenic poplars indicated a significantly increased *PmCesA2* transcript abundance along with cell wall thickening.

## Materials and Methods

### Source Plant

The biennial *Pinus massoniana* tree was propagated in the greenhouse of Nanjing Forestry University (NFU), in Jiangsu Province, China. Different tissues samples were directly frozen in liquid nitrogen and kept at -80°C before the RNA extraction. Total RNA was isolated from each sample using RNAprep Pure Plant Kit (Polysaccharides & Polyphenolics-rich) (Tiangen Biotech, Beijing, China) by following the manufacturer's instructions.

### Cloning of the *PmCesA2* Open Reading Frame

*PmCesA2* was cloned from a cDNA library constructed of RNA isolated from the needle tissue of *P. massoniana* using the Prime Script 1st Strand cDNA Synthesis Kit (Takara, Dalian, China). A pair of primers was designed according to the full-length coding region of the *CesA2* gene sequence of *P. taeda* (GenBank: AY789651.1). The PCR products were cloned into the pEASY-T1 (Transgen, Beijing, China) vector and transformed into *E. coli* DH5α, and then sequenced. All of the primers used in these assays are listed in **Supplementary Table S2**.

### Sequence and Phylogenetic Analyses

A total of 60 putative CesA protein sequences from various plant species were aligned using the ClustalW2 (http://www.ebi.ac.uk/Tools/clustalw2/index.html). A phylogenetic tree of CesAs protein family members of (*Arabidopsis thaliana*), (*Zea mays*), (*Oryza sativa*), (*Eucalyptus grandis*), (*Betula luminifera*), (*Populus trichocarpa*), (*P. taeda*), (*Pinus radiata*), (*Cunninghamia lanceolata*), and (*Picea glauca*) was constructed by the neighbor-joining method using MEGA7 with 1,000 replicates for the bootstrap analysis, and a 50% cutoff value (Kumar et al., 2016).

### *Agrobacterium*-Mediated Transformation of (P. Deltoides × P. Euramericana ‘Nanlin895’)

The binary vector pBI121 plasmid harboring the desired *CesA2* gene, where *PmCesA2* is under the control of the CaMV 35S constitutive promoter, was introduced into *Agrobacterium tumefaciens* strain EHA105 using the freeze–thaw method (Holsters et al., 1978). The *P. deltoides × P. euramericana* ‘Nanlin895’ leaf disks were inoculated with an infective suspension (OD_600_ = 0.7) of regenerated *A. tumefaciens*, under gentle shaking of 200 rpm for 1 h. Then, the leaf disks were dried using sterile paper towels and co-cultivated on MS medium (Murashige and Skoog, 1962) with 0.5 mg/L N-6-benzyladenine (6-BA), 0.004 mg/L thidiazuron (TDZ), 6 g/L agar, 25 g/L sucrose, 200 μM acetosyringone (AS), pH 5, and incubated in the dark at 28°C for 2 days (Mohammadi et al., 2018). This was followed by transferring the leaf disks to MS medium supplemented with 0.5 mg/L 6-BA, 0.004 mg/L TDZ, 6 g/L agar, 25 g/L sucrose, 400 mg/L cefotaxime, and 50 mg/L kanamycin, pH 5.8, under 16/8 h light/dark conditions at 23 ± 1°C in a phytotron to screen for the putative transformant explants. Afterward, the selected shoots were transferred to half-strength MS rooting medium; then, transferred to soil and propagated for complementary experiments. All the transgenic and wild type plants were acclimated and grown in the greenhouse at 18–23°C, 60% humidity, and with 18 h of light and 6 h of dark daily at the NFU.

### Plant Height and Biomass Measurements

Height from the basal stem to the peaks and stem diameter from 5 cm above the soil of 3-month‐old poplars were measured from each transgenic and WT lines. Fresh weight was immediately measured after sample collection. Then, the dry weight of the same plant material was determined after drying at 80°C for 48 h.

### Extraction of Chlorophyll Pigments

Total chlorophyll (TChl) content from the leaves of WT and transgenic plants was measured spectrophotometrically, by using 0.1 g of tissue ground in a pre-chilled mortar and pestle and extracted with 80% acetone (10 ml). After centrifugation at 10,000×*g*, the absorbance of a given extract was recorded at 663.8, 646.8, and 470.0 nm. The concentrations of chlorophyll a and b, and of total chlorophyll, were calculated following Lichtenthaler (1987).

### Determination of Cell Wall Composition

The 10th to 13th internodes of 3-month-old WT and transgenic plants used for cell wall composition analysis. To determine the cellulose content, 100-mg dried samples were degraded with a mixture of nitric and acetic acid (1:8, v/v, 30 min, 100°C) and centrifuged followed by dilution with 60% H_2_SO_4_. Cellulose levels were measured using a cold anthrone reagent at 620 nm (Jin et al., 2016) and the determination of lignin content carried out as previously described (Wang et al., 2017). The percentages of cellulose and lignin content were then averaged for three biological and technical replicates experiments. To determine the monosaccharide composition, 5 mg of the extract-free samples were extracted with 50 μl of sulfuric acid (72% w/w) at 37°C for 60 min, diluted with 4% H_2_SO_4_, autoclaved for 60 min, then allowed to cool to room temperature and an aliquot was neutralized with CaCO_3_. Analysis of monosaccharide composition was done using high performance liquid chromatography (Sluiter et al., 2008).

### PCR and Real-Time Quantitative PCR

Leaf tissues of all transgenic lines were collected for genomic DNA extraction from 1-month-old transgenic plants, by using the DNeasy Plant Mini Kit (Qiagen, Germany) following the manufacturer's instructions. The ensuing genomic DNA from each line was then used for PCR to confirm the integration of *PmCesA2*.

RNAprep Pure Plant Kit (Polysaccharides & Polyphenolics-rich) (Tiangen Biotech, Beijing, China) was used to extract RNA from the stem segment of WT and transgenic plant lines. The RNA was then used as a template in a reverse transcription reaction to produce cDNA, following the instructions of the PrimeScript RT Reagent Kit (Perfect Real Time) (TaKaRa Biotechnology, Dalian, China). QRT-PCR was used to assess the copy number and relative expression level of the *PmCesA2* gene (2^−ΔΔCT^) in the transgenic and WT lines using an ABI quantitative real-time RT-PCR system (Applied Biosystems, USA) and the SYBR Green PCR Master Mix according to the manufacturer's instruction. The relative expression levels of related *PmCesA2* genes were determined by same method. For the standard curve method cDNA was diluted (1,000-, 500-, 250-, 125-, and 62.5-fold) and two *PmCesA2* primers were used to amplify a product of 130 bp, and expression of the housekeeping gene *β-actin* primers was used for normalization expression to verify the real-time quantitative PCR reaction. All of the primers are listed in **Supplementary Table S2**.

### Southern Blot Analysis

Genomic DNA was extracted using cetyltrimethylammonium bromide method from WT plants and three transgenic poplar lines (Porebski et al., 1997). Approximately 10 μg of total genomic DNA was digested with EcoRI restriction enzyme, separated on a 0.8% agarose gel at 25 V overnight, and transferred onto Hybond N+ membrane. A 404-bp digoxin-labeled CaMV 35S was used as a probe for hybridization according to the instruction manual (DIG High Prime DNA Labeling and Detection Starter Kit I, Roche). Primers used for DIG labeling of CaMV 35S are listed in **Supplementary Table S2**.

### Scanning Electron Microscopy

The *Populus* stem (10th internode) of 3‐month‐old transgenic plants and the wild type were used for scanning electron microscopy (SEM), according to the previously described protocol by Yu et al. (2011) and the image analysis software IMAGEJ (https://imagej.nih.gov/ij/) was employed for quantifying morphological parameters of xylem cells (μm) and wall thickness.

### Statistical Analysis

All data for measured height, stem diameter, number of leaves, fresh weight, dry weight, chlorophyll content, carbohydrate content, cellulose, and lignin content were analyzed using the Student's *t*-test calculated in Microsoft Excel. Three biological replications with three technical replicates were performed each experiment. A one-way analysis of variance was applied to determine the significance of differences at *p* < 0.05.

## Results

### Cloning and Phylogenetic Tree Analysis

A 3,147 bp cDNA fragment was obtained by RT-PCR using the primers derived from the *CesA2* gene of *P. taeda*, encoding a protein of 1,057 amino acid residues, and having a molecular weight of 119.767 kDa and an isoelectric point of 8.4. Sequence analysis showed 98% similarity between the reported *CesA2* gene (AY789651.1) of *P. taeda* and *P. massoniana*.

**Figure 1:**
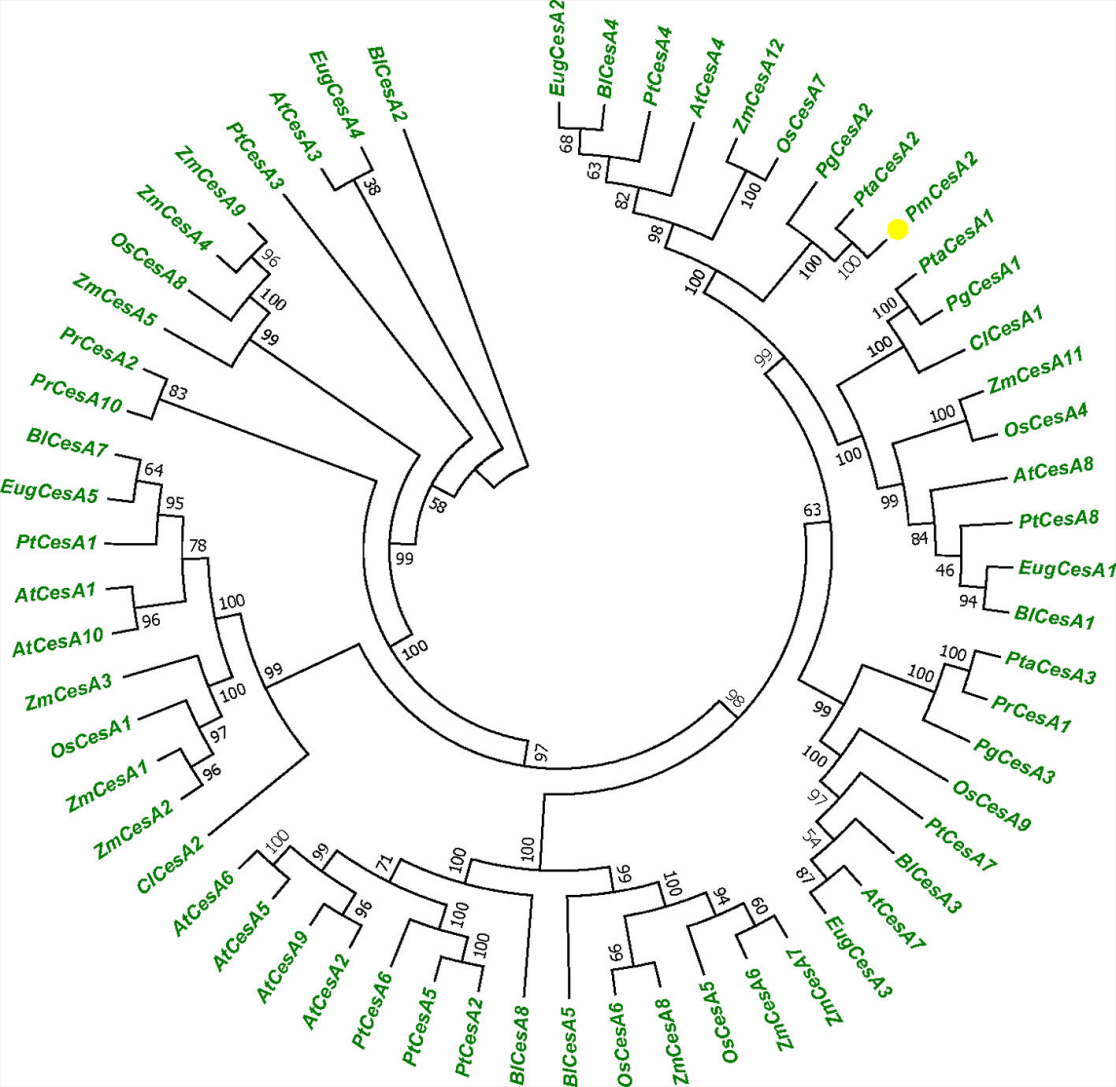
Phylogenetic tree showing relationships between the PmCesA2 amino acid sequence and other identified CesA amino acid sequences in different plant species. The unrooted tree was created with an alignment of 60 CesA protein sequences. The *Pinus massoniana CesA2* gene is shown with yellow triangle.

We used the 60 CESA protein sequences from plant species, *A. thaliana*, *P. trichocarp*a, *P. taeda*, and *Z. mays* to generate a phylogenetic tree (**Figure 1**). Protein sequence information was collected from NCBI database. To identify the species of origin for each CESA, the corresponding species name was included before each sequence name: *At*, *A. thaliana*; *Zm*, *Z. mays*, *Pt*, *P. trichocarpa*; *Pta*, *P. taeda*; *Pr*, *P. radiata*; *Cl*, *C. lanceolata*; *Pg*, *P. glauca*; *Eug*, *E. grandis*; *Os*, *O. sativa*; *Pm*, *P. massoniana*; and *Bl*, *B. luminifera.* Phylogenetic tree shows that *PmCesA2* in pine is ortholog of *PtCesA2* in poplar related secondary wall and the results confirmed the same function of these genes. All information about plant species and gene accession number are placed in **Supplementary Table S1**.

### Tissue-Specific Expression Analysis of *PmCesA2*

**Figure 2:**
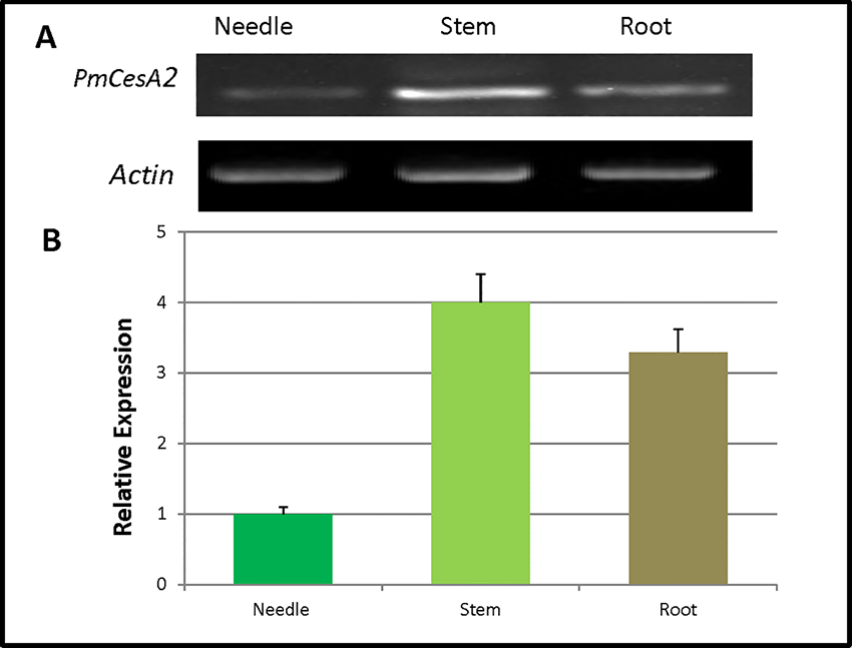
Tissue-specific expression of the *CesA2* gene in *Pinus massoniana*. **(A)** Tissue-specific expression pattern characterized by reverse transcription polymerase chain reaction (RT-PCR). **(B)** Tissue-specific expression pattern characterized by real-time quantitative PCR, for which expression levels were averaged from three replicates.

The quantitative real-time expression analyses of RNAs were performed to investigate the *PmCesA2* gene expression patterns in various tissues of *P. massoniana*. These RT-PCR results demonstrated that *PmCesA2* gene was expressed in all examined plant tissues (**Figure 2A**). In addition, the quantitative PCR (**Figure 2B**) analysis showed a high level of expression of *PmCesA2* in the stem and root than in the needle.

### Analysis of Expression and Integration of *PmCesA2 in* Nanlin895 Poplars

**Figure 3:**
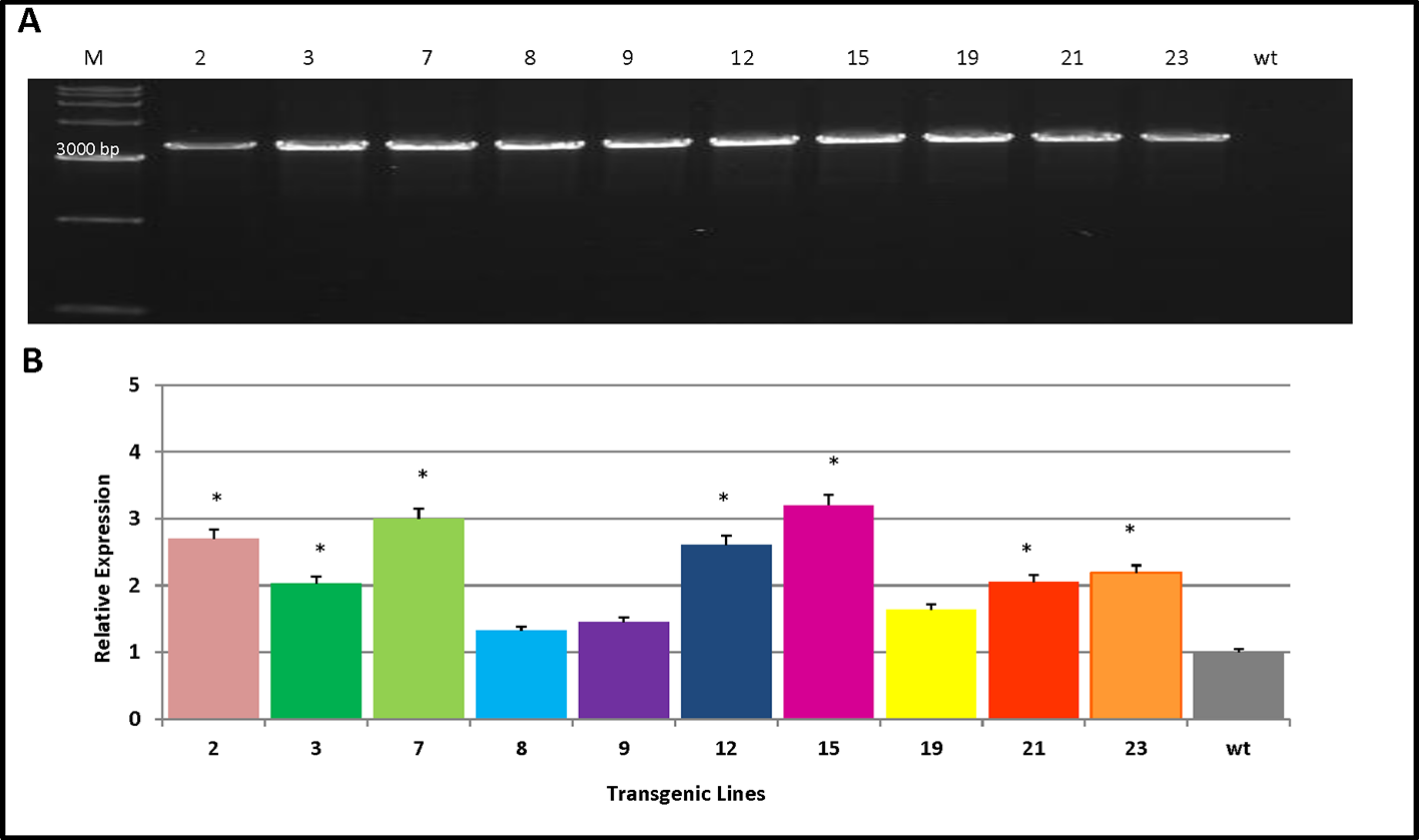
Verification of transgene integration and expression of *PmCesA2* into the poplar genome. **(A)** Integration of *PmCesA2* poplar transgenic lines using PCR amplification. Genomic DNA was extracted from the leaves of 1-month-old transgenic poplars. The PCR products were assessed through electrophoresis on 1.0% agarose gel. **(B)** Relative expression of *PmCesA2* in transgenic poplars by real-time PCR. Expressed levels were averaged ( ± SE) from three different samples per line. Actin served as the internal reference. * denotes significance at *p* < 0.05.

Ten transgenic poplar lines were verified by PCR analysis using the *PmCesA2*-specific primers. This indicated that the *PmCesA2* gene had been integrated into the genomes of 10 independent transgenic plant lines (**Figure 3A**). Relative expression levels of the *PmCesA2* gene were analyzed in 2-month-old stems of these verified transgenic lines, for which the quantitative real-time PCR analysis showed notable variation (**Figure 3B**). Specifically, *PmCesA2* expression levels in the L15 and L7 were relatively higher than in the other transgenic line and the wild type. The transgene copy numbers of *PmCesA2* were determined *via* Real time PCR based on formula *X* = *Y* – intercept/slope degrees (*X* = copy number, *Y* = Ct) (Movahedi et al., 2015). The results revealed that the average gene copy number of *PmCesA2* in transgenic plants is 7.45 with a slope of −3.36 and efficiency 0.994. Southern blot hybridization confirmed the stable integration of the *PmCesA2* into the genome of transgenic lines. Southern blotting analysis (**Supplementary Figure S1**) revealed that the transgene had integrated into the genome stably at two to three copies per genome. No bands were detected in the WT lanes.

### Growth and Morphological Characteristics in *PmCesA2* Transgenic Poplars

**Figure 4:**
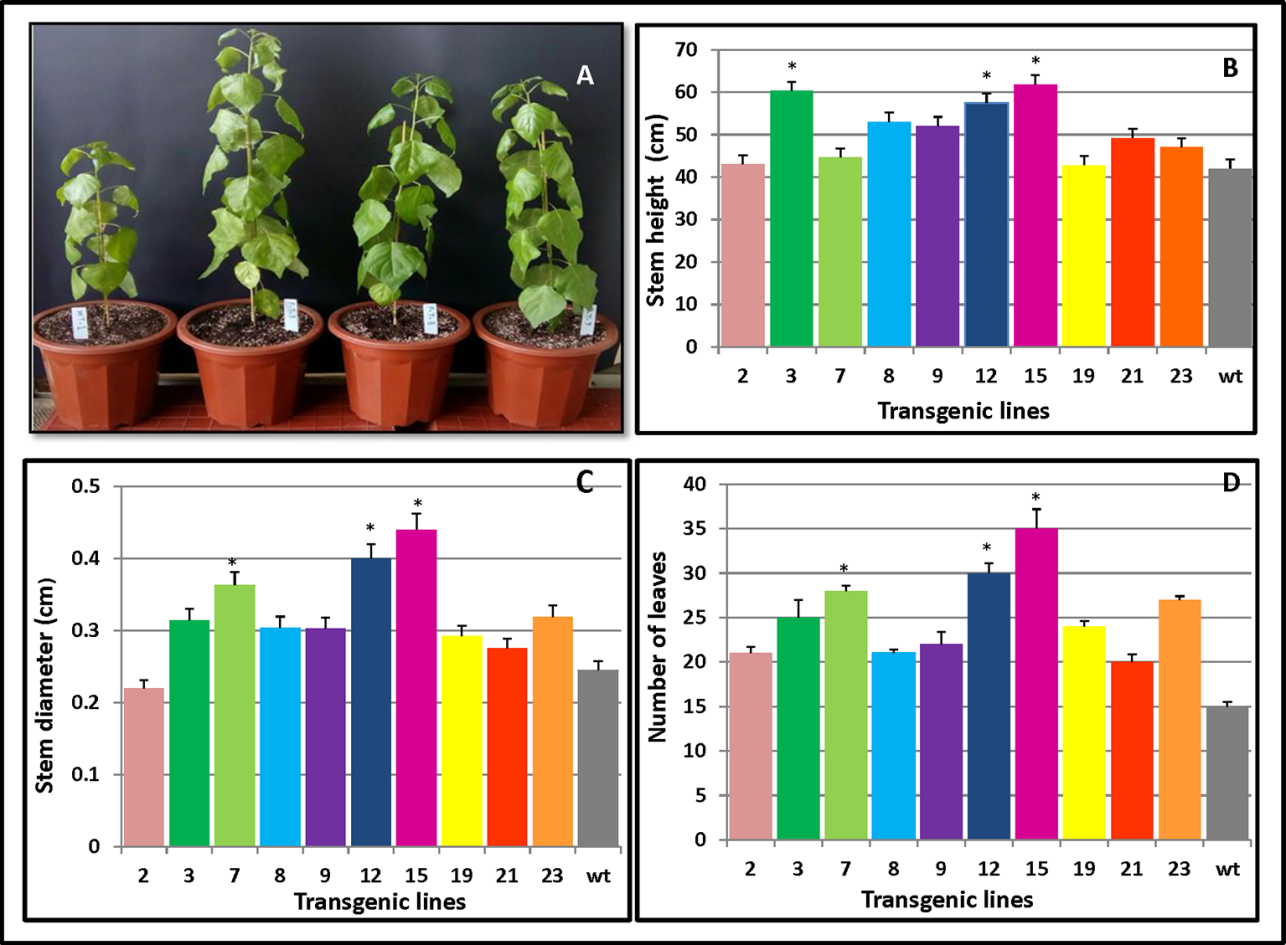
Phenotypic changes in *PmCesA2* transgenic lines. **(A)** Phenotypic comparison of 3-month-old poplar transgenic lines 9, 8, and 3 and the wild type (WT) (from right). Transgenic lines and WT plants compared for three growth traits: **(B)** heights, **(C)** stem diameters, and **(D)** number of leaves, averaged ( ± SE) from three different samples per line; * denotes significance at *p* < 0.05. The WT represents wild-type poplar while the others lines labeled with line numbers are of different *PmCesA2* poplar transgenic lines.

To investigate whether overexpression of *PmCesA2* could improve plant growth, we monitored the individual growth of three transgenic plants from each line and untransformed controls. Three months after planting into the soil, significant growth phenotype differences were observed between plants overexpressing *PmCesA2* and the wild type (WT). The former showed a fast‐growing phenotype with increased plant height, stem diameter, and leaf number (**Figure 4A**). Height measurements of the 10 lines clearly indicated that both L15 and L3 were significantly taller than WT (**Figure 4B**). With the exception of L2, which had a smaller stem diameter than WT, transgenic lines had a significantly greater stem diameter (31.83%) than did WT (**Figure 4C**). Similarly, concerning the number of leaves of the poplars, overexpression lines produced more leaves (48%) than did WT (**Figure 4D**). Generally, lines overexpressing the *CesA2* gene showed altered growth characteristics, demonstrating significantly increased height, stem diameter, and number of leaves.

**Figure 5:**
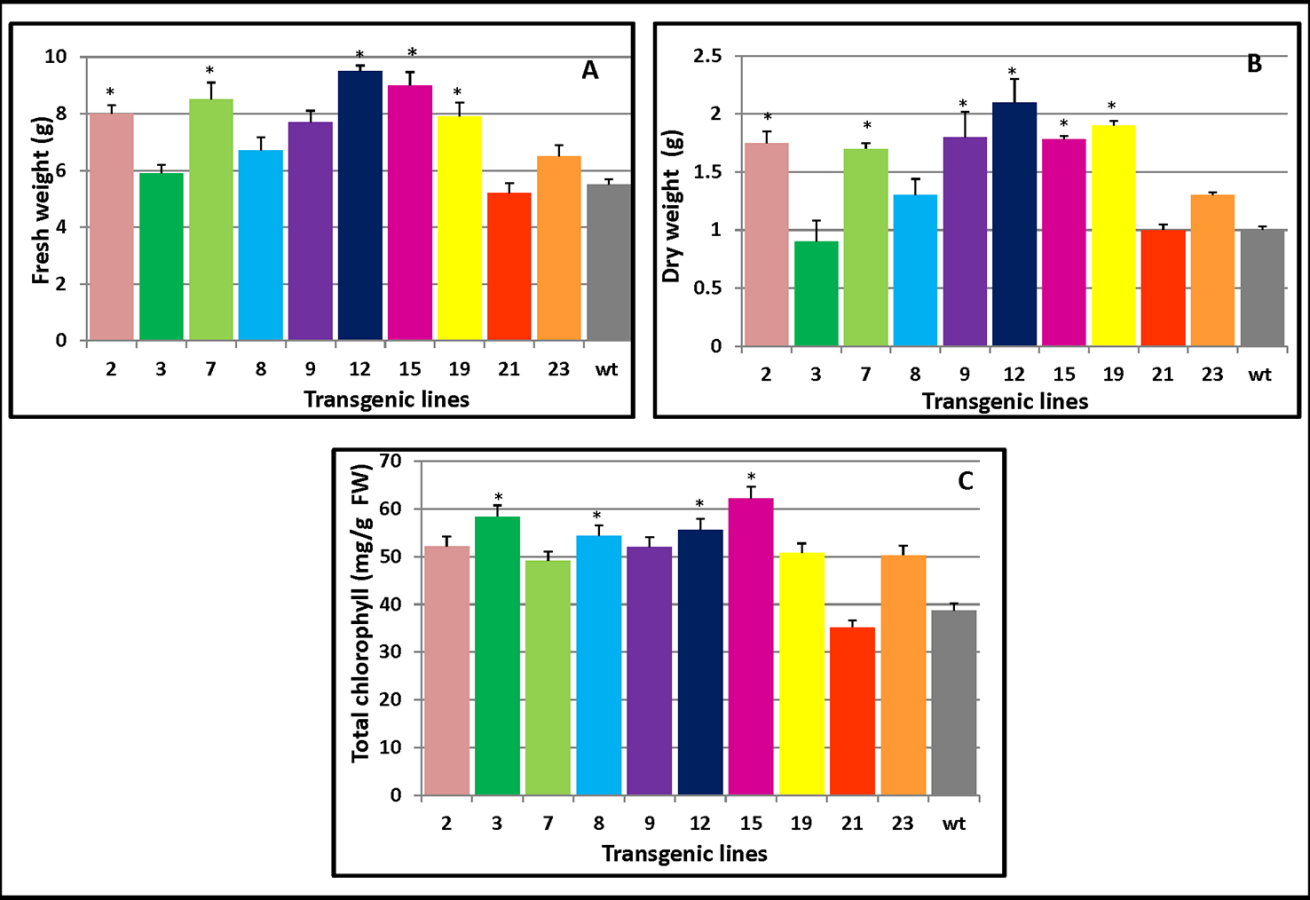
Changes in the biomass and chlorophyll contents of *PmCesA2* poplar transgenic lines. **(A)** Fresh weights and **(B)** dry weights of transgenic lines. **(C)** Chlorophyll contents of the leaves of different *PmCesA2* transgenic lines. All values are expressed as means ± SD (*n* = 3 biological replicates), * denotes significance at *p* < 0.05.

As **Figure 5A** clearly shows, compared with WT, the transgenic lines 15 and 12 both displayed high levels of fresh weight while lines 12 and 19 had the largest dry weight increase (**Figure 5B**). Both the fresh and dry weights of the transgenic plants exceeded those of WT plants which were 45% and 36% higher than WT, respectively. Apart from L21, which had lower chlorophyll content than the WT, the transgenic plants maintained higher chlorophyll contents (34.3%) than did WT (**Figure 5C**). The changes in the chlorophyll contents could increase photosynthesis and hence increase plant growth.

### Changes in Secondary Cell Wall Composition

SEM observations clearly showed a thickened secondary cell wall in transgenic line when compared with the WT. To investigate how the cell wall changed due to *PmCesA2* overexpression, the content of the cell wall's monosaccharide composition was analyzed in transgenic and wild-type plants. As **Table 1** shows, all transgenic lines showed significantly higher (up to 48%) xylose content with variations of other monosaccharides, compared to WT.

**Table 1:** Cell wall monosaccharide composition (mg g ^-1^) from stem of the control and *PmCesA2* transgenic plants.

Plant	Glucose	Xylose	Mannose	Galactose	Rhamnose	Arabinose
WT	35.16 ± 1.10	15.45 ± 0.36	2.83 ± 1.40	1.48 ± 0.19	0.67 ± 0.07	0.76 ± 0.18
L-2	36.05 ± 0.9	21.49 ± 0.78	2.19 ± 0.46	1.18 ± 0.09	0.66 ± 0.05	0.6 ± 0.03
L-3	35.59 ± 1.39	23.43 ± 0.60^*^	2.49 ± 0.53	1.33 ± 0.28	0.53 ± 0.11	0.61 ± 0.21
L-7	38.24 ± 1.22	22.56 ± 0.55	2.44 ± 0.29	1.16 ± 0.07	0.53 ± 0.13	0.71 ± 0.09
L-8	39.79 ± 2.16^*^	24.72 ± 2.63^*^	2.23 ± 0.78	1.19 ± 0.11	0.5 ± 0.05	0.58 ± 0.04
L-9	38.06 ± 1.20	24.4 ± 1.51^*^	2.53 ± 0.55	1.25 ± 0.17	0.6 ± 0.14	0.63 ± 0.06
L-12	39.71 ± 1.35^*^	25.61 ± 0.39^*^	2.83 ± 1.18	1.34 ± 0.10	0.65 ± 0.09	0.68 ± 0.23
L-15	37.19 ± 1.0	25.03 ± 1.01^*^	2.63 ± 0.74	1.35 ± 0.13	0.64 ± 0.11	0.81 ± 0.03
L-19	36.81 ± 1.25	22.64 ± 1.62	2.32 ± 0.51	1.17 ± 0.14	0.54 ± 0.08	0.6 ± 0.09
L-21	38.04 ± 1.20	22.63 ± 0.60	2.27 ± 0.43	1.2 ± 0.11	0.48 ± 0.12	0.57 ± 0.19
L-23	36.23 ± 1.40	22.79 ± 0.69	2.03 ± 0.15	1.09 ± 0.08	0.57 ± 0.07	0.6 ± 0.11

Data are the mean value ± SD of three biological replicates. *Denotes significance at p < 0.05.

**Figure 6:**
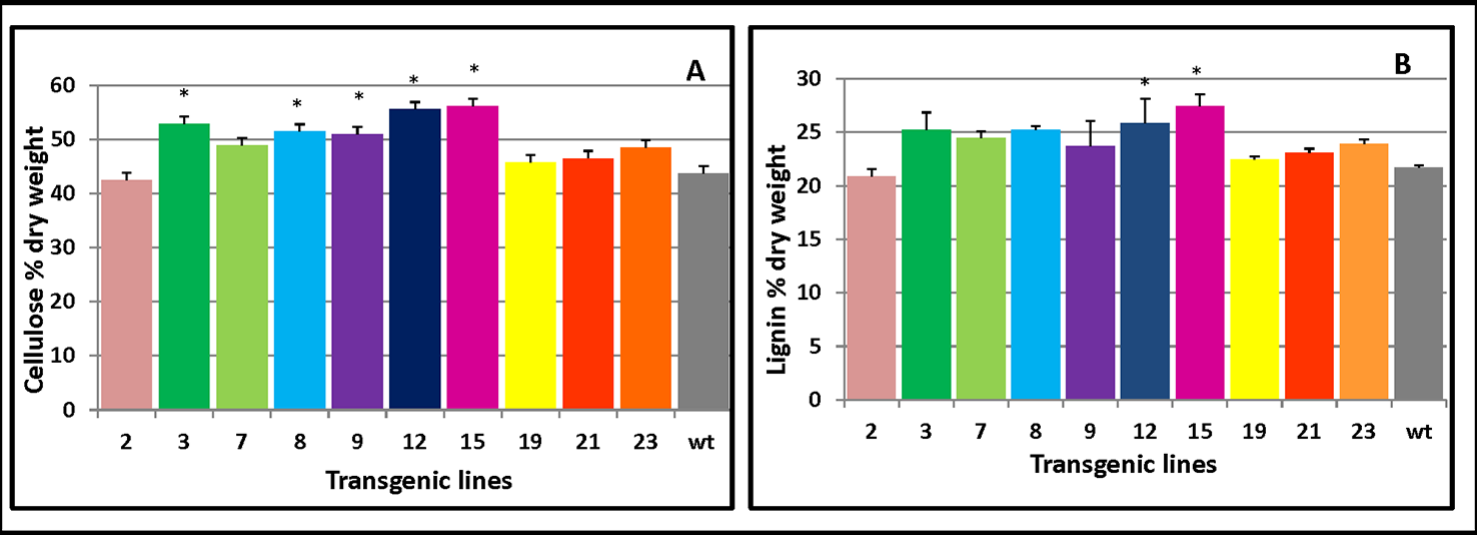
Cellulose and lignin content in *PmCesA2* transgenic lines. **(A)** Cellulose contents. **(B)** Lignin contents. All values are expressed as means ± SD (*n* = 3 biological replicates), * denotes significance at *p* < 0.05.

Cellulose production is correlated with the level of cellulose synthesis activity of *CesA* (Bringmann et al., 2012; Sánchez-Rodríguez et al., 2017). To assess which cell wall components drove the increased thickness of the secondary cell wall, cellulose and lignin content of transgenic stems were measured. The overexpressing lines contained more cellulose (**Figure 6A**) and lignin levels (**Figure 6B**) compared with the control plants. These results showed *PmCesA2* could enhance biomass yields in the transgenic plants due to high cellulose and lignin content.

### Changes in the Thickness of the Secondary Cell Walls in Transgenic *Populus*

**Figure 7:**
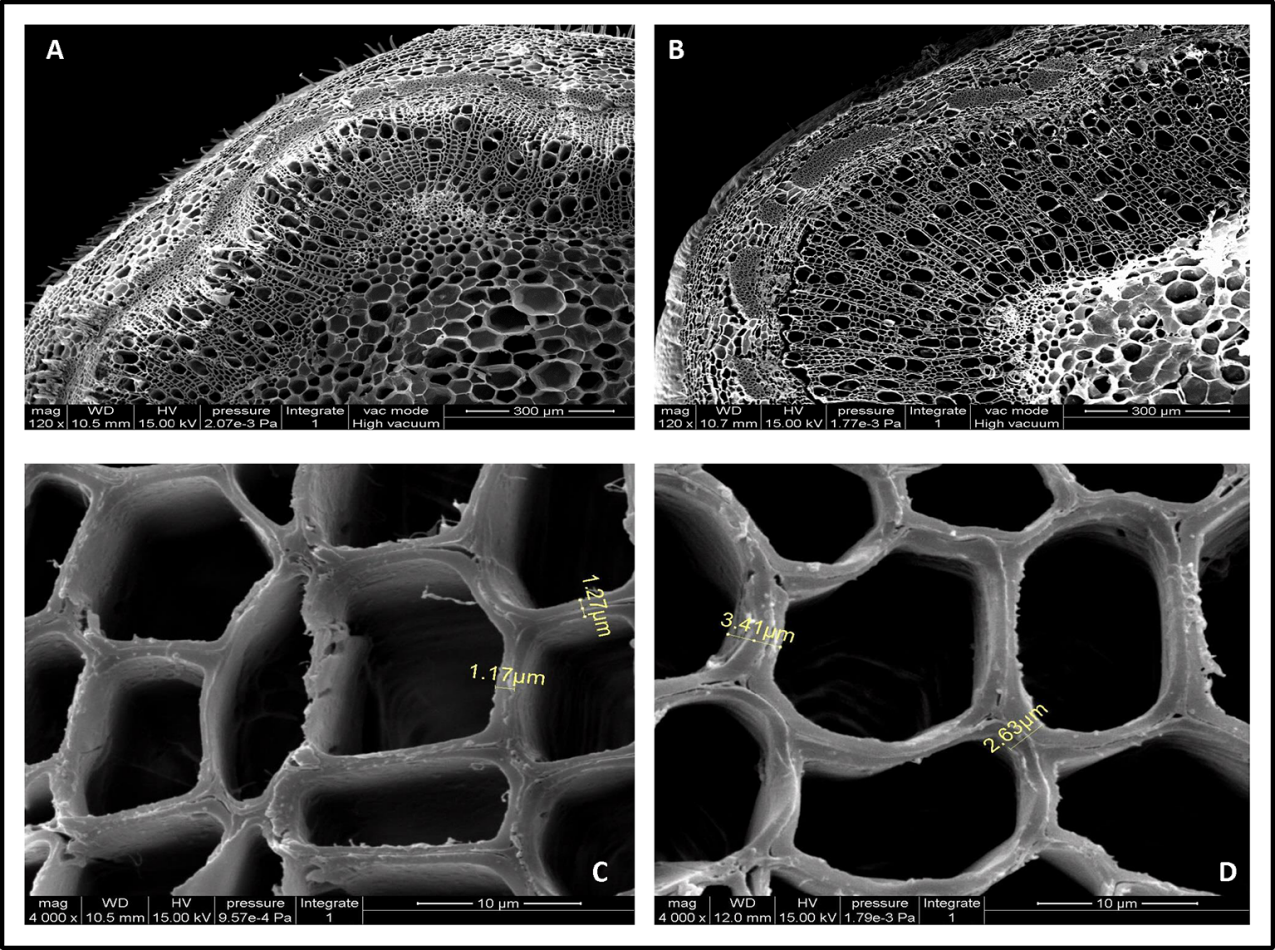
Scanning electron micrographs of the 10th internode of control and transgenic line plants. **(A, C)** are from wild-type poplar plants; **(B, D)** are from the transgenic line. Short yellow lines in **(C, D)** depict the difference between the cell wall thicknesses in the transgenic line and the wild-type plants. **(B)** Overexpression of *CesA2* displayed increased the number of secondary xylem cells compare with WT **(A)**.

Cell wall thickness arises from increased deposition levels of xylose and cellulose (Sun et al., 2014). To better understand the contribution of *PmCesA2* overexpression to secondary cell wall biosynthesis, microscopic analyses were conducted to measure the thicknesses with the stems of WT and transgenic plants. Notably, SEM images showed the entire cell wall had increased, including the secondary cell wall (at least twofold more), in the overexpressing lines when compared with that of WT (**Figure 7**). The xylem width of poplar transgenic lines had significantly increased compared to the wild-type plants (**Supplementary Table S3**). These results showed that overexpression of *PmCesA2* positively regulated the secondary cell wall formation in transgenic poplars.

### Alternation of Gene Expression in *PmCesA2* Transgenic Poplars

To determine whether *PmCesA2* impacts the expression of other genes involved in cellulose or lignin biosynthesis, we performed quantification analysis of the expression of cellulose and lignin biosynthetic genes in the stems of transgenic lines. Transcript abundance of *CesA5*, *CesA6* (two primary *CesA* genes), *Susy2* (key enzyme for secondary growth), and *PAL1*, *4CL1* (lignin biosynthesis genes) were up‐regulated in overexpressing lines compared to those in WT. Overexpression of *PmCesA2* gene could increase the expression levels of other primary wall *CesAs*, *Susy2* and lignin biosynthetic genes (**Supplementary Figure S2**).

## Discussion

The cell wall in plant cells provides structural support, underpinning plant growth and development (Leng et al., 2017). Cellulose, a major load-bearing structure of growing cell walls, has drawn much research attention for its various industrial applications. Therefore, researchers have tried to alter the process of cellulose biosynthesis to improve the growth, biomass production, and wood quality of plants. Here, we reported on the molecular and physiological behavior of transgenic poplar overexpressing *PmCesA2* under natural conditions. Over-expression of *PmCesA2* resulted in improved cellulose synthesis, plant growth, and biomass production in transgenic poplar lines compared to WT control plants, together with increased secondary wall thickening and width of the xylem. Although in plants, cellulose synthesis (*CesA*) genes have been shown to be fundamental for growth and development (Persson et al., 2007), in the last three decades, much effort has been met with limited success for improving cellulose synthesis through the overexpression of various the secondary *CesA_S_* (Burton and Fincher, 2014). Attempts to overexpress a secondary wall-associated *CesA* gene (*CesA7*) in *Arabidopsis*, (*CesA8*) in *Populus*, and (*CesA4*) in barley did not show any improvement in plant growth and plant biomass production (Zhong et al., 2003; Joshi et al., 2011; Tan et al., 2015). A recent study of *Panicum virgatum* highlighted that both *CesA4* and *CesA6* overexpression and knock-down to extreme levels in the transgenic lines resulted in decreased biomass production (Mazarei et al., 2018). In the present study, hybrid poplars overexpressing the structural *PmCesA2* gene from the source pine tree displayed considerable improvements in biomass production. In terms of cell wall composition, the overexpressing transgenic plants also showed higher cellulose and lignin levels. The present work therefore suggests that increases in cellulose and lignin led to enhanced biomass yields in these plants. In order to verify whether overexpression of *CesA2* could influence the transcription of carbohydrate metabolism and cellulose production, monosaccharide composition in the stem was studied. This observation showed that glucose and xylose were the most abundant sugars in all samples as expected and suggested that increased glucose content in the cell walls of transgenic lines compared with the corresponding wild-type could be ascribed to increases in the cellulose content. Compared with WT, we observed that the chlorophyll contents of all transgenic mature lines except one were significantly increased. The greatly increased chlorophyll contents would be expected to augment the photosynthetic capacity of plants which could potentially increase the biomass of the transgenic plants.

*EgCesA1,2,3* which are orthologous to *PtrCesAl*, *PtrCesA3*, and *PtrCesA2*, respectively, and *ZmCesA10*, *11*, *12* which are orthologous to *AtCesA4*, *AtCesA8*, and *AtCesA7*, respectively, all presented high expression in stem or stalk, tissues that undergo secondary cell wall biosynthesis in xylems (Appenzeller et al., 2004; Ranik and Myburg, 2006). Expression patterns of *PtaCesA1*, *PtaCesA2*, and *PtaCesA3* in loblolly pine are consistent with functional roles to their orthologous of secondary cell wall *CesA* genes in angiosperms which are highly expressed in developing xylem. *PtrCesA2* was isolated from a xylem cDNA library that exhibited a high degree of identity 82% with *AtCesA7* cDNA that has been associated with xylem development in *Arabidopsis* (Taylor et al., 1999), concluded that *PtrCesA2* from aspen is orthologous to *Arabidopsis AtCesA7*. Multiple alignments of full-length CesA protein sequences showed that secondary *CesAs* in pine are orthologs of *Arabidopsis* and poplar secondary wall CesAs (Nairn and Haselkorn, 2005). *PmCesA2* cDNA shows a high degree of similarity 98% with *PtaCesA2* cDNA that has been associated with secondary cell wall development in *P. taeda*. This indicates *PmCesA2* could be involved in secondary cell wall synthesis.

In this respect, it seems that growth improvement and biomass production were achieved *via* genetic manipulation, at least for poplar trees. Cellulose biosynthesis and secondary wall thickness of *Arabidopsis* are affected by mutations in each of the secondary *CesAs* (*CESA4/IRX5*,* CESA7/IRX3*,**and *CESA8/IRX1*), leading to collapsed xylem phenotype (Turner and Somerville, 1997; Taylor et al., 1999; Taylor et al., 2000; Taylor et al., 2003). Mutations in each of the primary *CesAs* can lead to reduced organ growth, which has been interpreted as the consequence of growth anisotropy being lost (Pagant et al., 2002; Fujita et al., 2013; Chen et al., 2016). *CesA5* and *CesA2* are responsible for secondary wall cellulose biosynthesis in *Arabidopsis* seed coat epidermis (Mendu et al., 2011). Using various methods, both *in vitro* and *in planta*, it was shown that the primary wall *CesAs* interacts with other secondary wall *CesAs*, thus raising the possibility that mixed complexes of primary and secondary wall structure *CesAs* could occur at particular times (Carroll et al., 2012). Overexpression *PmCesA2* gene enhances the expression of other primary wall *CesAs* as well as changes in expression of gene related to cell growth, cellulose (*Susy2*) or lignin (*PAL1* and *4CL1*) production.

In conclusion, our results demonstrate the overexpressing of the *PmCesA2* gene is directly relevant to plant growth and development in poplar due to enhanced cellulose synthesis which led to a thickened secondary cell wall. Based on our results, we propose that the *CesA2* genes' overexpression may cause to enhance the expression of other genes linked to cell growth and cellulose production in transgenic plants. Our approach could serve as an efficient biotechnological modification tool for producing enhanced plant biomass.

## Data Availability Statement

This article contains previously unpublished data. The name of the repository and accession number(s) are not available.

## Author Contributions

SM and KM designed and directed the project. SM, KM, and AM performed the experiments. SM and FW processed the experimental data. SM and KM wrote the manuscript with input from all authors. KJ supervised the project. All authors discussed the results and commented on the manuscript.

## Conflict of Interest

The authors declare that the research was conducted in the absence of any commercial or financial relationships that could be construed as a potential conflict of interest.
