# Supplementary material for: Increase in Cell Wall Thickening and Biomass Production by Overexpression of PmCesA2 in Poplar
Source: Front Plant Sci. 2020 Feb 20;11:110. doi: 10.3389/fpls.2020.00110 (PMC7044265; doi:10.3389/fpls.2020.00110)
Supplement: Supplementary file 1 [file DataSheet_1.pdf]

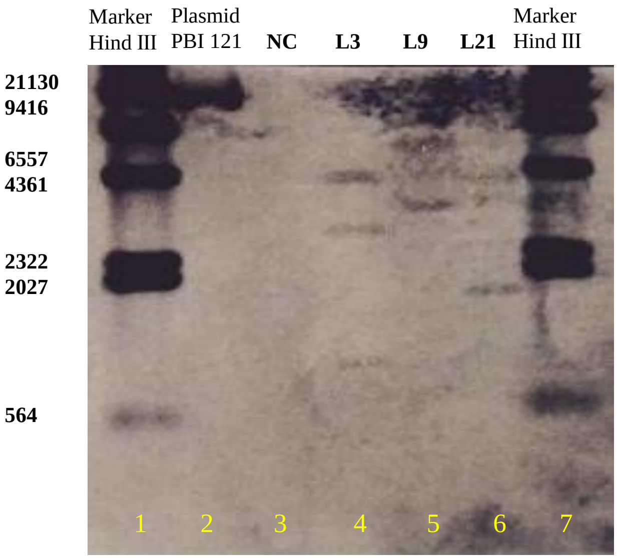

Figure S1. Southern blot analysis of transgene (*PmCesA2*). Lane 1: Marker, Lane 2: *PBI121:CesA2* plasmid, Lane 3: Negative Control (DNA of untransformed plant), Lane 4–6: genomic DNA extracted from three transgenic poplar lines. Lane 7: Marker.
