# Supplementary material for: Increase in Cell Wall Thickening and Biomass Production by Overexpression of PmCesA2 in Poplar
Source: Front Plant Sci. 2020 Feb 20;11:110. doi: 10.3389/fpls.2020.00110 (PMC7044265; doi:10.3389/fpls.2020.00110)
Supplement: Supplementary file 2 [file DataSheet_2.pdf]

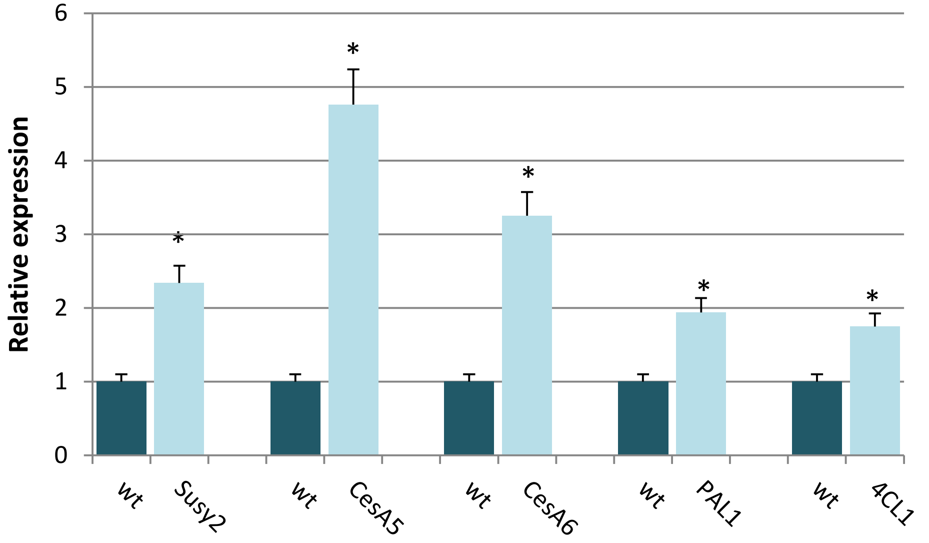

Figure S2. Relative transcript levels of *Susy2, CesA5*, *CesA6*, *PAL1*, and *4CL1*. Actin was used as the internal control. All values are expressed as means ± SD; (n = 3 biological replicates), * denotes significance at $p < 0.05$.
