# Supplementary material for: Increase in Cell Wall Thickening and Biomass Production by Overexpression of PmCesA2 in Poplar
Source: Front Plant Sci. 2020 Feb 20;11:110. doi: 10.3389/fpls.2020.00110 (PMC7044265; doi:10.3389/fpls.2020.00110)
Supplement: Supplementary file 3 [file Table_1.doc]

| Accession Number Name | Accession Number Name |
| --- | --- |
| | AAQ63935 | PrCesA10 | | --- | --- | | AAQ63936 | PrCesA2 | | AAT57672 | PrCesA1 | | AAX18647 | PtaCesA1 | | AAX18648 | PtaCesA2 | | AAX18649 | PtaCesA3 | | AFM37965 | ClCesA1 | | AFM37966 | ClCesA2 | | BT106827 | PgCesA2 | | BT116636 | PgCesA1 | | BT116976 | PgCesA3 | | O48946 | AtCesA1 | | O48947 | AtCesA2 | | XP_024459034.1 | PtCesA1 | | XP_002310629.1 | PtCesA2 | | XP_024463935.1 | PtCesA3 | | XP_002301856.2 | PtCesA4 | | XP_002310628.1 | PtCesA5 | | XP_002307145.1 | PtCesA6 | | XP_006381880.1 | PtCesA7 | | XP_002316815.1 | PtCesA8 | | ACJ38664.1 | BlCesA1 | | ACJ38665.1 | BlCesA3 | | ACJ38666.1 | BlCesA4 | | ACJ38667.1 | BlCesA5 | | Q84M43 | OsCesA2 | | Q84ZN6 | OsCesA8 | | Q94JQ6 | AtCesA6 | | Q851L8 | OsCesA5 | | | Q2IB39 | EugCesA5 | | --- | --- | | Q2IB40 | EugCesA4 | | Q2IB41 | EugCesA3 | | Q2IB42 | EugCesA2 | | Q2IB43 | EugCesA1 | | Q5JN63 | OsCesA4 | | Q6AT26 | OsCesA1 | | Q6YVM4 | OsCesA6 | | Q8L778 | AtCesA5 | | Q8LPK5 | AtCesA8 | | Q9AV71 | OsCesA7 | | Q9LLI1 | ZmCesA9 | | Q9LLI2 | ZmCesA8 | | Q9LLI3 | ZmCesA7 | | Q9LLI4 | ZmCesA6 | | Q9LLI5 | ZmCesA5 | | Q9LLI6 | ZmCesA4 | | Q9LLI8 | ZmCesA2 | | Q9LLI9 | ZmCesA1 | | Q9SJ22 | AtCesA9 | | Q9SKJ5 | AtCesA10 | | Q9SWW6 | AtCesA7 | | Q67BC7 | ZmCesA12 | | Q67BC8 | ZmCesA11 | | Q69P5 | OsCesA9 | | Q84JA6 | AtCesA4 | | Q941L0 | AtCesA3 | | AGV22110.1 | BlCesA8 | | AGV22109.1 | BlCesA7 | | AGV22107.1 | BlCesA2 | |

**Table S1.** Accession numbers of sequences utilized in the phylogenetic tree.
