# Supplementary material for: Increase in Cell Wall Thickening and Biomass Production by Overexpression of PmCesA2 in Poplar
Source: Front Plant Sci. 2020 Feb 20;11:110. doi: 10.3389/fpls.2020.00110 (PMC7044265; doi:10.3389/fpls.2020.00110)
Supplement: Supplementary file 4 [file Table_2.doc]

**Table S2.** List of primers used in this study.

| **Analysis** | **Name** | | **Forward and Reverse primers** |
| --- | --- | --- | --- |
| PCR | *CesA2* | | F (5`-ATGGAGGCCAAGGCGGGA-3`)  R (5`- GGCCCCATTCTTCAGCAATGT-3`) |
|  | *G-CesA2* | | F (5`-GGAAAAAGCAACCATATAAAACTATTGCC-3`)  R (5`- TGCATAATTACATTCTTACAGTACCAGGC-3`) |
| Quantitative  Real-Time  PCR | *CesA6* | | F (5'- AATTCCCACACAAGGGGAACTTGATT-3')  R (5'- TGAACTCTATTTCCATGACTCATGTGTG-3') |
|  | *CesA5* | | F (5'-TTGTTAAGCATGAAGGAGGACATGAC-3')  R (5'-TGTACGGGTTTATCTTGCTTGAGGG-3') |
|  | *Susy2* | | F (5'-GACATTTGCTGAGGATGCAGCC-3')  R (5'-CTGTGTGACACCCATCTTGTAAGC-3') |
|  | *CesA2* | | F (5′-ACTTGAAGGTTACGAAGAACATGAG-3')  R (5′- TACAGACTCTGGAACACCACCA-3') |
|  | *PAL1* | | F (5´-CTTGGAAGCAATTACCAAGCTACTT-3')  R (5´-ACTTCTCCGTTGGGACCAGTG-3') |
|  | *4CL1* | | F (5´-AGCAAAACAAGCCAAAGCCTCAAA-3')  R (5´-CTGTGTTAGCTCTGAAAAGTGCAAGC-3') |
|  | *β-actin* | | F (5′-CCACGAAACTACTTACAACTCCATC-3')  R (5'-GGGCTGTGATTTCCTTGCTC-3′) |
| Southern blot probes | *CaMV 35S* | F (5′- GGCCATGGAGTCAAAGATTCAAATAGA-3′)  R (5′- AACGTCTTCTTTTTCCACGATGCTC-3′) | |
