# Supplementary material for: Increase in Cell Wall Thickening and Biomass Production by Overexpression of PmCesA2 in Poplar
Source: Front Plant Sci. 2020 Feb 20;11:110. doi: 10.3389/fpls.2020.00110 (PMC7044265; doi:10.3389/fpls.2020.00110)
Supplement: Supplementary file 5 [file Table_3.doc]

**Table S3**. Radial width of xylem and cell wall thickness in stems of control and *PmCesA2* transgenic poplar plants. Data are the mean value ± SD of three biological replicates. * denotes significance at *p* < 0.05.

| Samples | Radial width of xylem (μm) | Wall thickness (μm) |
| --- | --- | --- |
| **WT** | 1835 ± 34.1 | 1.20 |
| **L 15** | 3651 ± 78.04* | 3.01* |
| **L 7** | 2584 ± 57.76* | 2.68* |
| **L 12** | 2872 ± 61.58* | 2.86* |
